# Associations between hurricane stressors, social capital, and climate anxiety among individuals who experienced Hurricanes Helene and Milton

**DOI:** 10.1371/journal.pmen.0000749

**Published:** 2026-10-13

**Authors:** Francis Dalisay, Wenlin Liu, Masahiro Yamamoto, Haoran Chu, Jay Hmielowski, Young-Rock Hong, Jiawei Liu, Benjamin K. Johnson

**Affiliations:** 1 College of Journalism and Communications, University of Florida, Gainesville, Florida, United States of America; 2 Department of Communication, University at Albany, State University of New York, Albany, New York, United States of America; 3 School of Medicine, Department of Family and Preventive Medicine, Emory University, Atlanta, Georgia, United States of America; University of Santo Tomas College of Science, PHILIPPINES

## Abstract

This study examined how hurricane stressors and three forms of social capital—bonding, bridging, and linking—are associated with climate anxiety, and whether the three forms of social capital would moderate the relationship between hurricane stressors and climate anxiety. We analyzed data from a cross-sectional survey (*N* = 1,342) administered in Georgia, North Carolina, and South Carolina following Hurricane Helene and in Florida following Hurricane Milton in 2024. Results showed that hurricane stressors were positively associated with climate anxiety, and these associations held after controlling for symptoms of generalized anxiety. Findings also revealed a nuanced role of social capital. Bonding social capital had a direct negative association with climate anxiety. However, our results suggested that bonding social capital’s protective role is conditional and reverses under high stress. That is, bonding social capital weakened the relationship between hurricane stressors and climate anxiety only when hurricane stressors were low. As stressors accumulated, bonding social capital strengthened the association between stressors and climate anxiety. In contrast, both bridging and linking social capital were positively associated with climate anxiety and strengthened the positive relationship between hurricane stressors and climate anxiety. We discussed theoretical insights and practical implications.

## 1. Introduction

The Southeastern region of the United States experienced the compounded destructive effects of Hurricanes Helene and Milton during the fall of 2024 [1,2]. The intensities of both hurricanes have been linked to climate change [3]. In the aftermath of these storms, news media reported that, beyond physical destruction, Hurricanes Helene and Milton had significant negative impacts on survivors’ mental health [4,5]. These accounts are consistent with past studies providing compelling evidence that extreme weather events, including hurricanes, impose significant psychological strain, including heightened anxiety [6,7]. Prior research particularly demonstrates that individuals who experience hurricane stressors—such as personal losses, injuries, or other disruptions—may face an increased risk of developing climate anxiety [8], a form of distress distinct from generalized anxiety and defined as emotional or psychological distress marked by heightened fear and constant worrying about the climate crisis [9].

Against the backdrop of studies examining climate anxiety, another line of research has highlighted the protective role of social capital, or social connections [10], in mitigating distress associated with natural disasters [11]. Indeed, a long-established area of disaster research suggests that social capital could shape how communities respond to climate-related hazards and influence their capacity for recovery [12,13]. Despite this evidence, little is known about how social capital relates to climate anxiety, or whether social capital may moderate the psychological effects of hurricane stressors.

To bridge the above-noted research gaps, the present study seeks to accomplish three goals. First, within the context of the recovery from Hurricanes Helene and Milton, we assess the relationship between hurricane stressors and climate anxiety. Second, we examine the relationship between social capital and climate anxiety. Third, we explore whether social capital moderates the relationship between hurricane stressors and climate anxiety. We analyze data from online surveys conducted in late 2024 with residents of U.S. Federal Emergency Management Agency (FEMA)-declared disaster counties in Georgia, North Carolina, and South Carolina, following Hurricane Helene, and in Florida following Hurricane Helene/Milton.

## 2. Literature review

### 2.1 Hurricane stressors and climate anxiety

The present study is guided by three theoretical frameworks to explain the relationship between hurricane stressors and climate anxiety: the stress process model, the environmental identity framework, and the concept of eco-grief.

This stress process model provides a framework that explains the relationships between stressors, resources, and mental health outcomes [14]. According to this model, stressors, which are acute or chronic disruptive events (e.g., adversities due to a hurricane), trigger mental health responses, such as increased anxiety. The model also views resources as central in explaining how a stressor affects mental health outcomes. That is, the extent to which a stressor impacts one’s mental health could be conditioned by the resources one has available to cope with the stressor. As we will discuss below, resources facilitated by social ties could play a role in moderating the relationship between hurricane stressors and climate-induced anxiety.

Based on the stress process model, we first predict that hurricane stressors would be positively associated with climate anxiety. As mentioned, hurricane stressors refer to the adversities and critical losses of resources that one experiences due to a hurricane [15]. Such stressors can range from losing access to resources such as food, water, shelter, or electricity to experiencing injuries. Climate anxiety is a form of environment-related distress specifically tied to the perceived threat of climate change [16], manifesting as extreme and persistent concern and rumination about climate change [9]. A recent study conducted by Asgarizadeh et al. showed that personal past experiences with climate-related events was associated with heightened levels of anxiety [17].

Yet we note that direct experiences with hurricane stressors could plausibly produce generalized anxiety. As stated, research has specifically established a positive relationship between experiencing stressful hurricane events and increased levels of psychological distress [8,18], including anxiety [19,20]. For instance, in their study of individuals who experienced Hurricane Katrina, Galea et al. found that hurricane-related stressors, namely, experiencing property loss, physical injury, and physical adversity, were positively associated with symptoms of anxiety [6].

However, climate anxiety represents a theoretically and empirically distinct outcome. Generalized anxiety is characterized by diffuse, object-independent excessive worry about a number of events, disproportionate to the actual likelihood or impact of those events [21]. Climate anxiety, on the other hand, is object-specific and ecologically grounded and reflects an appraisal of systemic environmental threat rather than a pathological response to personal danger. Indeed, Clayton and Karazsia’s [9] work has demonstrated empirically that climate anxiety is distinct from general psychological distress, establishing it as a separate construct warranting independent investigation. In a 5-year cohort survey of Texas and Florida residents who directly experienced category 3 or higher hurricanes, Garfin and Wong-Parodi found that having previous direct exposure to hurricanes, measured with items such as having a home destroyed, losing property or a pet, and knowing someone who was injured, was positively associated with climate anxiety [8].

Complementing the stress process model, the framework of environmental identity [22] and the concept of ecological grief [23] provide the psychological mechanism for why directly experiencing hurricane stressors activates this climate-specific form of anxiety rather than generalized distress. First, on a cognitive level, environmental identity (EID) represents a sense of connection to the non-human natural environment influencing how individuals perceive and act toward the environment [22,24]. For people with strong EID, the natural environment plays an important role in their self-concept and who they are. Having a strong EID is positively linked with pro-environmental attitudes and behaviors [22], as well as climate change risk perception and climate anxiety [25]. While EID varies across individuals, the EID framework suggests that in processing the destruction resulting from a major hurricane, people may interpret such an event through an ecological framework. That is, through an ecological lens, hurricane-related stressors such as losses, damages, and adversities may not be perceived solely as personal misfortune. Rather, experiencing such stressors may serve as evidence of a larger, systemic environmental threat posed by climate change, thereby activating climate-specific anxiety rather than generalized anxiety. Furthermore, through an ecological lens, the losses and damages experienced because of an extreme weather event or natural disaster, such as a major hurricane, may manifest affectively as ecological grief, which refers to grief felt in response to experienced or anticipated ecological losses [23]. As grief itself has been known to associate with anxiety, researchers have found that ecological grief is associated with eco-related anxiety [23,26]. In sum, environmental identity and ecological grief provide the psychological mechanism explaining why hurricane stressor experience produces climate anxiety as a distinct outcome rather than undifferentiated psychological distress.

Based on the literature above, we propose the following hypothesis:

**H1:** Hurricane stressors will be positively associated with climate anxiety.

### 2.2 Social capital

Our study also examines the potential relationship between social capital and climate anxiety. Social capital refers to social ties and informal or formal connections that enable individuals to develop norms of trust, reciprocity, and honesty [10]. People with higher social capital tend to be more likely to engage in collective action, ranging from joining or volunteering for community organizations to watching over their neighbors’ properties [10,27]. Scholars have identified three forms of social capital: bonding, bridging, and linking. Bonding, or homophilous social capital, refers to the strong ties one has with kin and close friends [28]. Such ties offer emotional support and build shared identity, although they tend to form among those who already share similar values and goals. In contrast to bonding social capital, bridging and linking social capital are both considered heterophilous, as they involve ties among individuals of diverse groups [28]. Bridging and linking social capital are typically facilitated through participation in political, civic, religious, and work-related activities. Such activities lean toward being goal-oriented and offer networking opportunities and access to external resources via extra-community ties [28]. However, bridging and linking social capital are conceptually distinct. Bridging social capital involves horizontal connections between people from different groups and social levels, while linking social capital represents vertical connections between people of varying levels of power (e.g., being connected to public officials).

Social capital may serve a protective role against distress experienced because of disasters. For instance, a study conducted in Japan during the recovery from the 2011 Fukushima nuclear power plant disaster showed that higher levels of social capital were associated with experiencing less anxiety [11]. The central mechanism underlying social capital’s protective role against distress is its capacity to facilitate the support that enables individuals to cope with a disaster. For example, Hawkins and Maurer [29] analyzed social capital during the recovery period following Hurricane Katrina. They found that bonding social capital effectively fostered immediate support after the hurricane. That is, during the period immediately after Hurricane Katrina, most individuals who experienced the storm first received or lent help to their families or others in their immediate networks. Moreover, bridging and linking social capital, which connect individuals from different backgrounds, could facilitate access to resources needed to recover from a natural disaster [29]. Bridging and linking social capital could serve as sources for instrumental support, or tangible resources, after a disaster such as a hurricane [29]. Examples of instrumental support in the context of hurricane recovery include receiving water, food, shelter, or financial assistance. Linking social capital, in particular, which encompasses connections between people of varying levels of power or authority (e.g., knowing a government official), could open access to resources that one would not otherwise have available [28]. Having access to resources is important when navigating through or coping with stressful experiences. After a hurricane, those with connections to government officials or institutions may be the first to know where to look for governmental aid or how to apply for disaster relief. In this case, as Hawkins and Maurer’s work suggests, bridging and linking social capital may be more important than bonding social capital for sustaining the long-term post-hurricane recovery and revitalization of a community [29].

However, it is important to acknowledge that a line of work informed by Portes indicates that social capital may have a “dark side” [30]. That is, it may result in negative consequences in relation to disaster resilience and mental health [31–33]. For instance, disaster literature points to the “contagion of stress” effect of social capital among close-knit communities [31]. Specifically, in cohesive communities, the stress experienced by one individual can easily spread through a well-connected social network, creating a contagion that then amplifies the entire community’s anxiety levels [33]. Bonding social capital, which is based on strong ties shared among homogenous group members, may potentially heighten anxiety about climate change if such ties include individuals who already feel extremely anxious about the climate crisis. Thus, individuals within bonding social networks may also face similar challenges and experience similar levels of climate anxiety due to the homogeneity of such networks. Additionally, higher bridging and linking social capital increases the potential of being exposed to a wide range of information regarding climate change, particularly, those pertaining to climate threats. As previous research has found, exposure to information about the impacts of climate change is associated with heightened levels of climate anxiety [16].

In sum, the research to date indicates that social capital can serve a protective role against distress [11,28,29]. However, a specific line of research suggests social capital may function as a double-edged sword, exerting both positive and negative effects on disaster resilience [32] and health outcomes [33]. In acknowledging the positive and negative aspects of social capital, we propose the following:

**RQ1:** How are (a) bonding, (b) bridging, and (c) linking social capital differentially associated with climate anxiety?

We also examine the possibility that social capital would moderate the proposed relationship between hurricane stressors and climate anxiety. As previously stated, the stress process model views resources as central in explaining how a stressor affects mental health outcomes [14]. This model suggests that resources, such as those facilitated through social capital, would condition the effects that stressors have on mental health outcomes [15]. First, on the one hand, extant research supports a “buffering” potential of social capital. That is, it could help mitigate various forms of psychological distress and enhance mental health [34,35]. Such research specifically suggests that social capital markedly buffers the distress associated with experiencing a disaster [11,36]. An explanation for social capital’s “buffering” potential is that it fosters the support and resources necessary to cope with a natural disaster.

On the other hand, we also noted the “dark side” potential of social capital [30]. That is, it is possible that the stresses experienced because of a hurricane may be amplified by social capital, which would then result in heightened climate anxiety. Disaster literature points to the “contagion of stress” effect of social capital among close-knit communities [31]. For instance, in cohesive communities, the stress experienced by one individual can easily spread through a well-connected social network, creating a contagion that then amplifies the entire community’s anxiety levels [33].

In sum, the above literature suggests that social capital can both buffer and amplify the relationship between hurricane stressors and climate anxiety. However, the empirical evidence on how different types of social capital—bonding, bridging, or linking—can differently shape climate anxiety or moderate the psychological impact of hurricane stressors on anxiety, remains significantly under-explored. Instead of hypothesizing a uniform buffering effect, we propose the following research question:

**RQ2:** How will (a) bonding, (b) bridging, and (c) linking social capital moderate the proposed positive association between hurricane stressors and climate anxiety?

## 3. Materials and methods

### 3.1 Ethics statement

The survey and study were approved by the Institutional Review Board (IRB) for human subjects research based at the University of Florida, Gainesville, FL, USA [IRB Protocol # ET00043626, Approval date: October 10, 2024] prior to data collection. Electronic informed consent, specifically, active consent via a click-through process, was ensured before participants were allowed to complete the survey. That is, prior to completing the online survey, participants read a cover page that informed them about the survey and study’s purpose, the voluntary nature of the study, and that their responses would remain anonymous. The bottom of the informed consent page contained instructions stating that if participants agreed to participate, they could click on the “I agree to participate” button to continue; however, if they did not consent to participate, they could click on the “I do not wish to participate” button or just close the browser window.

### 3.2 Procedure and participants

This cross-sectional study utilized online surveys targeting residents of counties with FEMA disaster declarations in Georgia, North Carolina, and South Carolina following Hurricane Helene and in Florida following both Helene and Milton. We contracted Qualtrics to collect the data and administer the survey. Quota sampling was used to ensure representation across demographic characteristics, including age and sex. In Georgia, North Carolina, and South Carolina, surveys were administered in late October 2024, roughly one month after Hurricane Helene made landfall, through the end of November 2024 (recruitment of participants commenced on October 30, 2024, and ended on November 28, 2024). In Florida, the survey was administered through November 2024, about a month after Hurricane Milton made landfall (recruitment of participants commenced on November 9, 2024, and ended on November 21, 2024). In total, there were 3,329 completion attempts. Participants who did not consent to taking the survey, sped through the survey, or indicated that they did not live in a county impacted by either of the hurricanes, were excluded. This left a final sample size of *N* = 1,342. The completion rate was 40%.

### 3.3 Measures

#### 3.3.1 Hurricane stressors.

We asked participants whether, in the past month, they had indirectly or directly experienced hurricane-related stressors from Hurricane Helene/Milton. We listed the following six stressors: (a) lack of any resource (food, water, shelter, electricity) for over a week, (b) loss of sentimental possessions or pets, (c) self or household member had health problems, (d) financial loss, (e) housing situation changed as a result of the hurricane, and (f) experienced any issues with mold, contamination, or structural damage in your home due to the hurricane. The first four items were adapted from Lowe et al. [15] and we created the two remaining items. Responses to all the items for hurricane-related stressors were scored with 1 = yes, 0 = no. We summed and combined the items to form a single measure (*M* = 2.32, *SD* = 1.91, α = .77).

#### 3.3.2 Climate change anxiety.

Three items from Clayton and Karazsia’s climate anxiety scale were used [9]: (a) thinking about climate change makes it difficult for me to concentrate, (b) I have nightmares about climate change, and (c) my friends say I think about climate change too much [9]. Responses were measured along a 5-point scale (1 = strongly disagree, 5 = strongly agree), then we averaged and combined the items to form a single measure (*M* = 2.47, *SD* = 1.07, a = .85).

#### 3.3.3 Social capital.

Drawing on prior work on personal social capital, online/social-media-enabled social capital, bonding and bridging social capital, and linking social capital in disaster recovery, we adapted items to assess three forms of social capital relevant to post-disaster coping and resource access [29,37–39]. Bonding social capital captured access to close interpersonal support, bridging social capital captured access to resources and information beyond one’s immediate family or close friends, and linking social capital captured perceived access to government-related contacts, information, and disaster-relief resources.

*Bonding* social capital was measured with the following items: (a) I have people I can turn to and ask for help, (b) there is someone I can talk about my problems with, and (c) there is someone who can give me emotional support (1 = strongly disagree, 5 = strongly agree). The items were averaged and combined to form a single measure (*M* = 3.79, *SD* = .98, α = .88).

*Bridging* social capital was measured with three items: (a) I know someone with resources that my family or friends do not have, (b) there are people I can turn to for new job opportunities, and (c) I know someone who can help me learn about things that happen outside my community (1 = strongly disagree, 5 = strongly agree). We averaged and combined these items to form a single measure (*M* = 3.29, *SD* = .94, α = .74).

*Linking* social capital was measured with the following three items: (a) I know someone who works in the government, (b) I know how to apply for disaster relief from the government, and (c) I know where to look for governmental aid during disasters (1 = strongly disagree, 5 = strongly agree). We averaged and combined the items to form a single measure (*M* = 3.08, *SD* = .95, α = .66).

#### 3.3.4 Covariates.

We measured and controlled for age, biological sex, state (Florida, Georgia, North Carolina, South Carolina), educational attainment (continuous), income (continuous), race and ethnicity, and political orientation (1 = very conservative, 5 = very liberal). For state, Florida was treated as the reference group in all the regression models as discussed in our analytical strategy below. We thus included only Georgia, North Carolina, and South Carolina as covariates in the regression models. For race, White was treated as the reference group, and only Non-White Hispanic or Latino, Black, Asian and other ethnicities as covariates in our regression models. We also included a measure for generalized anxiety as a covariate [40], which included the following four items adapted from the GAD-7 that asked respondents whether in the last 2 weeks, how often they had experienced the following: (a) been bothered by feeling nervous, anxious, or on edge, (b) been bothered by not being able to stop or control worrying, (c) been bothered by worrying too much about different things, and (d) been bothered by having trouble relaxing. Responses were averaged and combined to form a single measure (*M* = 2.17, *SD* = .96; α = .93). Finally, the bonding, bridging, and linking social capital were included as covariates when they were not respectively entered as moderators.

### 3.4 Data analysis strategy

All analyses were performed using SPSS version 30 and the regression analyses were performed using the PROCESS macro [41]. Listwise deletion was used to deal with missing data, and as such, all analyses were performed on complete data (*n* = 1,327). To test H1 and investigate RQ, we used PROCESS Model 1 to estimate three separate moderation analyses, each respectively examining one form of social capital (bonding, bridging, and linking social capital) as a moderator of the hurricane stressor-climate anxiety relationship. This approach allowed us to isolate the unique moderating effect of each social capital type while controlling for the others. Ninety-five percent bootstrap confidence intervals (CIs) with 5,000 resamples were generated for inference tests. The hypothesized moderation effects were assessed at the analytical mean and one standard deviation below and above the mean of each social capital variable. All predictors (hurricane stressors and the three social capital variables) were mean-centered prior to analysis for the purpose of interpreting the correlation when the interaction term was added in the model. The covariates previously identified were included in all regression analyses. We note that prior to analyzing the data, we also inspected the zero-order correlation between the measure of generalized anxiety and climate change anxiety, which was *r* = .24 (*p* < .05). This level of correlation does not raise concerns about multicollinearity. Lastly, all regression coefficients reported below are unstandardized (*b*).

## 4. Results

### 4.1 Sample characteristics

Participant characteristics are reported in Table 1. The mean age of participants was 46.03 years (*SD* = 17.33). There were also slightly more males (55.1%) than females (44.9%), a majority of the sample resided in Florida (53.5%), and most identified as White (63%) and politically moderate (39.3%). Finally, the median education level reported was “one or more years of college,” and the median annual income reported was between “$40,000-$59,999.”

**Table 1:** Means, standard deviations, and descriptive statistics of key variables among participants (*N* = 1,342).

	Descriptives	
Sex	Male 55.1%, Female 44.9%	
Age	46.09 (17.32)	
State	Florida (53.5%) Georgia (11.7%) North Carolina (20.7%) South Carolina (14%)	
Education	Median “one or more years of college”	
Income	Median $40,000-$59,999	
Ethnicity	White (63%) Black (21.5%) Non-White Hispanic or Latino (4.8%) Asian (1.4%) Other (10%)	
Political orientation	Very conservative (16.8%) Conservative (20.8%) Moderate (39.3%) Liberal (14.5%) Very liberal (8.6%) 2.77 (1.15)	
	**M(SD)**	**Cronbach’s α**
Hurricane stressor	2.33 (1.91)	.77
Climate change anxiety	2.47 (1.07)	.85
**Moderators**		
Bonding social capital	3.79 (.98)	.88
Bridging social capital	3.29 (.94)	.74
Linking social capital	3.08 (.95)	.66

### 4.2 Hypothesis and research questions

H1 expected that experiencing hurricane stressors would be positively associated with climate anxiety. This hypothesis was supported. Our results indicate that with all other variables simultaneously taken into account, hurricane stressors were positively associated with climate anxiety in all three regression models reported in Table 1 (*b* = .073, *se* = .031, *p* < .001; *b* = .065, *se* = .016, *p* < .001; *b* = .065, *se* = .016, *p* < .001).

RQ1 investigated how the three forms of social capital—bonding, bridging, and linking—are associated with climate anxiety. As shown in Table 2, there was a negative association between bonding social capital and climate anxiety across all three regression models (*b* = -.178, *se* = .032, *p* < .001; *b* = -.179, *se* = .032, *p* < .001; *b* = -.180, *se* = .032, *p* < .01). However, in all three regression models, bridging (*b* = .220, *se* = .036, *p* < .001; *b* = .217, *se* = .036, *p* < .001; *b* = .212, *se* = .036, *p* < .01) and linking social capital (*b* = .229, *se* = .031, *p* < .001; *b* = .230, *se* = .032, *p* < .001; *b* = .237, *se* = .031, *p* < .01) were found to be both positively associated with climate anxiety.

**Table 2:** Associations between hurricane stressors and social capital (bonding, bridging, and linking) with climate anxiety.

	Bonding SC as Moderator		Bridging SC as Moderator		Linking SC as Moderator	
	b	sr² (%)	b	sr² (%)	b	sr² (%)
Age	−.010 (.002)***	1.77%	−.010 (.002)***	1.97%	−.010 (.002)***	1.98%
Sex	−.044 (.050)	.04%	−.041 (051)	.04%	−.031 (.050)	.02%
Education	.000 (.015)	.00%	−.001 (.015)	.00%	−.002 (.015)	.00%
Income	.055 (.012)***	1.22%	.056 (.012)***	1.24%	.054 (.012)***	1.18%
Location						
Georgia	−.072 (.084)	.04%	−.060 (.084)	.03%	−.062 (.084)	.03%
North Carolina	−.086 (.071)	.08%	−.070 (.071)	.05%	−.063 (.071)	.04%
South Carolina	−.251 (.076)**	.58%	−.232 (.077)**	.50%	−.228 (.076)**	.48%
Race						
Hispanic	.096 (.126)	.03%	.110 (.126)	.04%	.106 (.125)	.04%
Black	.221 (.068)**	.56%	.222 (.068)**	.57%	.222 (.068)**	.57%
Asian	.231 (.213)	.06%	.235 (.213)	.07%	.224 (.213)	.06%
Other	.079 (.085)	.05%	.094 (.086)	.07%	.090 (.085)	.06%
Political orientation (5 = liberal)	.036 (.022)	.14%	.037 (.022)	.15%	.039 (.022)*	.17%
Generalized anxiety	.178 (.031)***	1.78%	.182 (.031)***	1.88%	.180 (.031)***	1.82%
Hurricane stressors	.073 (.016)***	1.12%	.065 (.016)**	.88%	.065 (.016)***	.90%
Bonding social capital	−.178 (.032)***	1.71%	−.179 (.032)***	1.71%	−.180(.032)***	1.74%
Bridging social capital	.220 (.036)***	1.91%	.217 (.036)***	1.91%	.212 (.036)***	1.83%
Linking social capital	.229 (.031)***	2.91%	.230 (.032)***	2.91%	.237 (.031)***	3.10%
Bonding social capital × stressors	.047 (.013)***	.72%	–	–	–	–
Bridging social capital × stressors	–	–	.053 (.013)***	.84%	–	–
Linking social capital × stressors	–	–	–	–	.062 (.014)***	
R² (%)	29.52%		29.64%		29.92%	

*N* = 1327. * *p* < .05, ** *p* < .01, *** *p* < .001. Outcome is climate anxiety. Entries are unstandardized regression coefficients with standard errors in parentheses. The squared semipartial correlation (sr²) for each predictor is also reported, expressed as a percentage, and reflects the unique variance accounted for by each predictor.

RQ2 investigated whether the association between experiencing hurricane stressors and climate anxiety was moderated by the three forms of social capital. First, as shown in Table 2, an interaction term between hurricane stressors and bonding social capital was significant (*b* = .047, *se* = .013, *p* < .001), indicating that the association between hurricane stressors and climate anxiety varied at different levels of bonding social capital. As shown in Table 3, the relationship between hurricane stressors and climate anxiety was significant when bonding social capital was at its mean (*b* = .073, *se* = .016, *p* < .001) and 1SD above the mean (*b* = .119, *se* = .021, *p* < .001), but not at 1SD below the mean (*b* = .027, *se* = .020, *p* = .176). Fig 1 shows the plot of this interaction effect. At low levels of hurricane stressors, participants with high bonding social capital reported a relatively low level of climate anxiety. However, as hurricane stressors increased, the slope for those with high bonding social capital became much steeper than those with lower bonding social capital.

**Table 3:** A moderation model of bonding, bridging, and linking social capital on climate change anxiety.

	*Effect*	*SE*	*t*	*p*	*LLCI*	*ULCI*
Bonding SC -1 *SD*	.027	.020	1.35	.176	-.012	.065
Bonding SC *M*	.073	.016	4.56	.000	.041	.104
Bonding SC + 1 *SD*	.119	.021	5.67	.000	.078	.160
Bridging SC -1 *SD*	.015	.021	.732	.464	-.026	.056
Bridging SC *M*	.065	.016	4.05	.000	.033	.096
Bridging SC + 1 *SD*	.114	.020	5.83	.000	.075	.152
Linking SC -1 *SD*	.007	.021	.316	.752	-.034	.048
Linking SC *M*	.065	.016	4.10	.000	.034	.100
Linking SC + 1 *SD*	.123	.020	6.22	.000	.085	.162

Bootstrap sample size- LL = lower limit; UL = upper limit; CI = bias corrected 95% bootstrap confidence interval.

**Fig 1:**
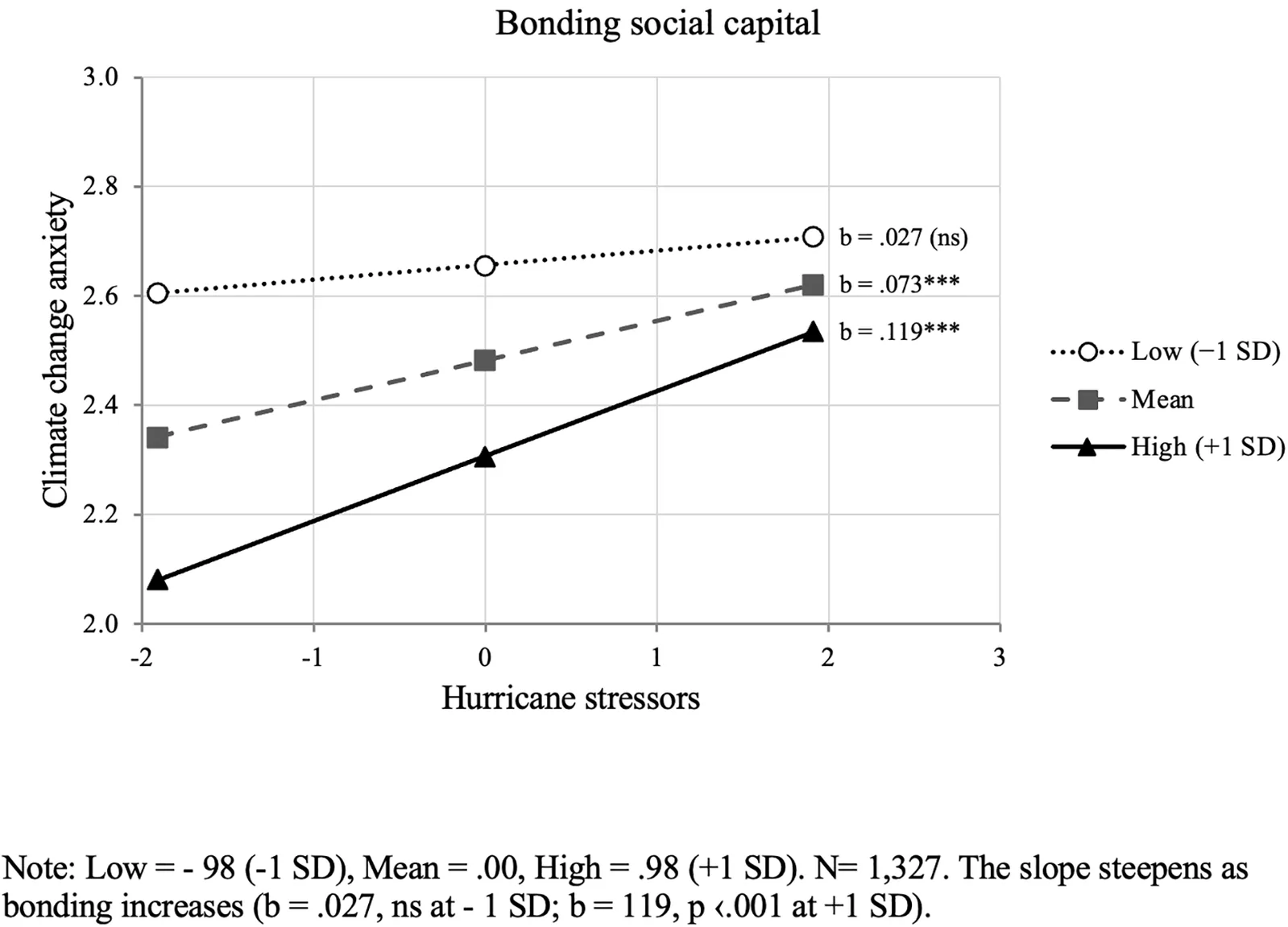
Bonding social capital as a moderator of the relationship between hurricane stressors and climate change anxiety.

Second, as Table 2 shows, the interaction between hurricane stressors and bridging social capital was significant (*b* = .053, se = .013, *p* < .001). Similar to what was found for bonding social capital, the relationship between hurricane stressors and climate anxiety was not significant when bridging social capital was at 1SD below the mean (*b* = .015, *se* = .021, *p* = .464). Yet, as Table 3 indicates, this relationship became progressively stronger as levels of bridging social capital increased from its mean (*b* = .065, *se* = .016, *p* < .001) to 1SD above the mean (*b* = .114, *se* = .020, *p* < .001). The plot for this interaction is illustrated in Fig 2. In contrast to the results for bonding social capital, Fig 2 indicates that at lower levels of hurricane stressors, climate anxiety was slightly higher for participants with higher bridging social capital. As hurricane stressors increased, the slope of the stress-anxiety relationship became steeper for participants with higher levels of bridging social capital. In contrast, the slope was relatively flat for those with low bridging social capital.

**Fig 2:**
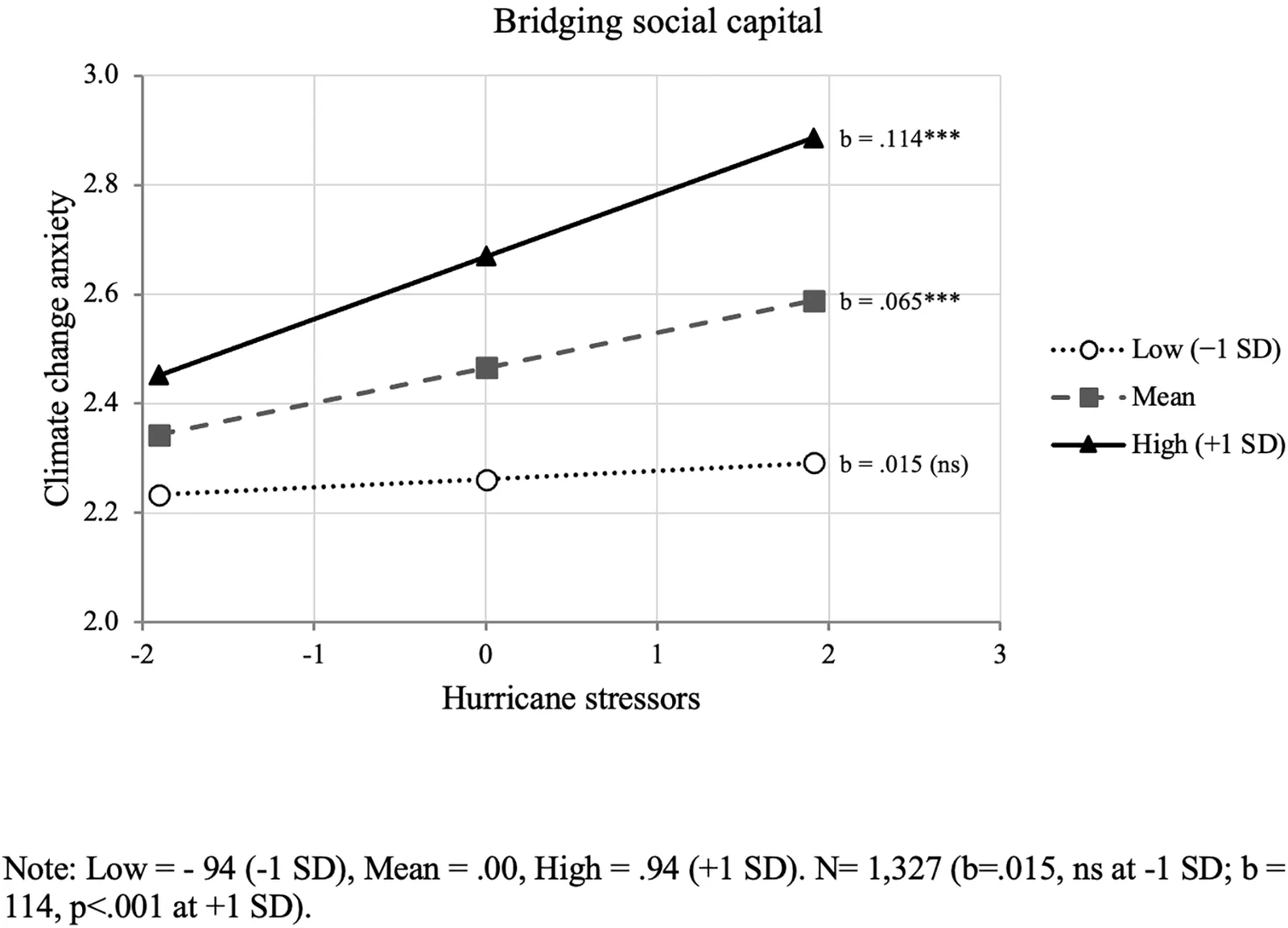
Bridging social capital as a moderator of the relationship between hurricane stressors and climate change anxiety.

Third, the interaction between hurricane stressors and linking social capital was also significant (*b* = .062, *se* = .014, *p* < .001), and similar to the pattern found for both bonding and bridging social capital, the relationship between hurricane stressors and climate anxiety was not significant when linking social capital was at 1SD below the mean (*b* = .007, *se* = .021, *p* = .752). Yet, the relationship became stronger as levels of linking social capital increased from its mean (*b* = .065, *se* = .016, *p* < .001) to 1SD above the mean (*b* = .123, *se* = .020, *p* < .001) (see Table 3 and Fig 3). As shown in Fig 3, the pattern of the plot for linking social capital is similar to that for bridging social capital. That is, at lower levels of hurricane stressors, climate anxiety was slightly higher for participants with higher linking social capital, and the slope for the relationship between hurricane stressors and climate anxiety was steeper for participants with higher linking social capital.

**Fig 3:**
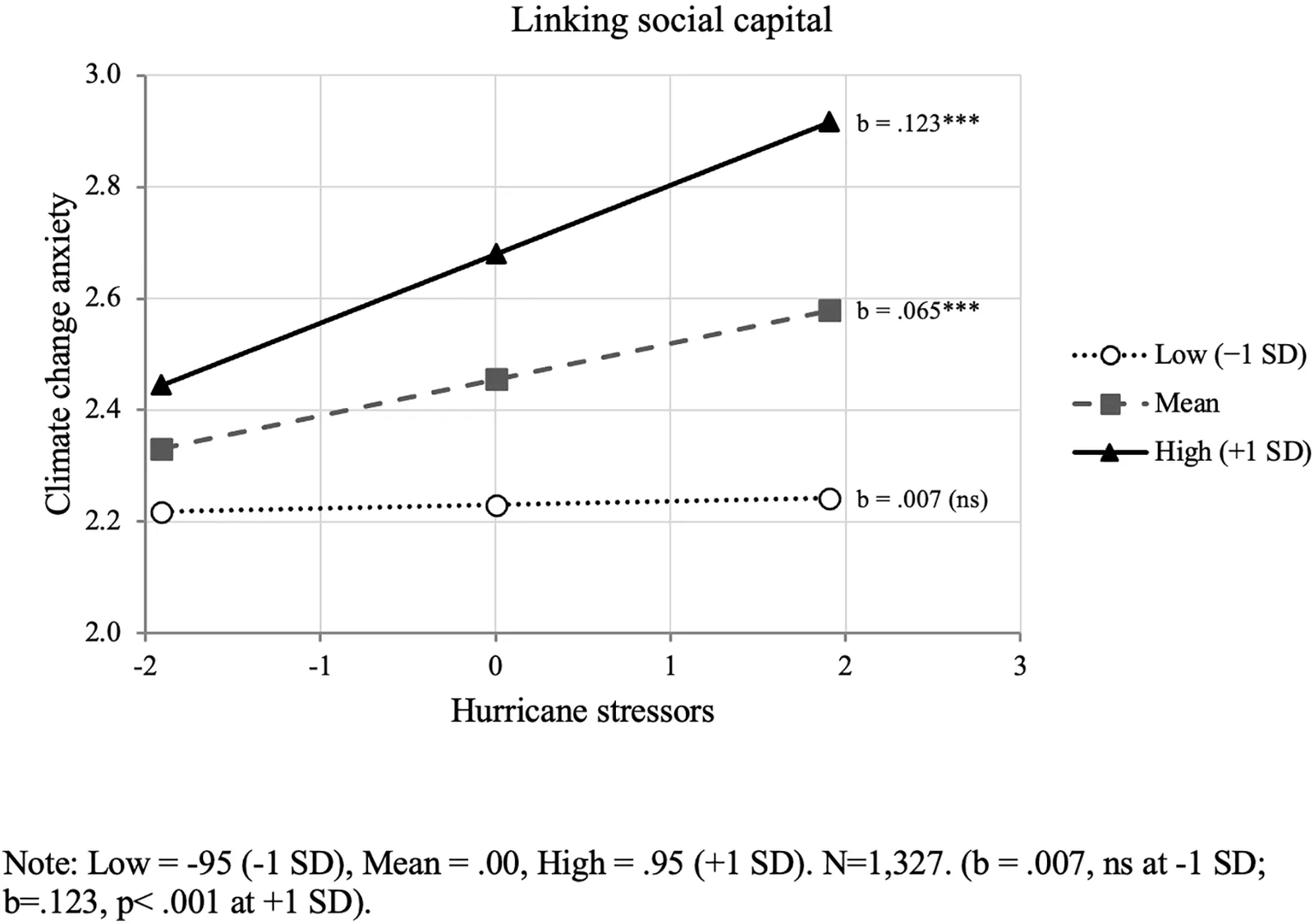
Linking social capital as a moderator of the relationship between hurricane stressors and climate change anxiety.

### 4.3 Ancillary analyses: Association of covariates with climate anxiety

As Table 2 shows, income (b = between .054 to.056) and black race/ethnicity (b = between .221 to .222) were both positively associated with climate anxiety, and South Carolina residence (relative to the Florida reference group) was negatively associated (b = between -.251 to .228). Also, generalized anxiety as a covariate was positively associated with climate change anxiety (b = between -.182 to -.178).

## 5. Discussion

Hurricanes Helene and Milton caused widespread devastation across several Southeastern states in the U.S. [1,2]. The first purpose of the present study was to test whether stressful experiences or stressors associated with Hurricanes Helene and Milton would be positively related with climate anxiety. We analyzed data from surveys conducted in the counties of four U.S. states impacted by the two hurricanes. Our finding confirms that hurricane stressors were positively associated with climate anxiety, supporting previous research conducted by Garfin and Wong-Parodi [8] and more broadly expanding past research linking personal experiences with hurricanes to psychological distress. This finding is also consistent with the stress process model [14], which explains why stressors such as hurricane-related losses and damages are associated with adverse psychological outcomes. Moreover, the positive association between hurricane stressors and climate anxiety aligns with the psychological mechanisms proffered by the environmental identity [22] framework and the concept of ecological grief [23], which both suggest that experiences with hurricane stressors could be interpreted through an ecological lens connecting immediate loss to the broader systemic threat of climate change. However, we note that our surveys did not include measures for environmental identity and ecological grief. Thus, future studies could examine these variables as potential mechanisms that may explain the association between hurricane stressors and climate anxiety. In addition, climate change risk perceptions or climate change attributions are also constructs that reflect cognitive appraisals of climate change as a threatening phenomenon [42], and as such are conceptually similar to climate anxiety. As our study did not assess these constructs, future research could investigate how hurricane stressors may be linked to such variables.

The second purpose of our study was to explore the relationship between social capital and climate anxiety. We examined three forms of social capital: bonding, bridging, and linking. Our findings, on the one hand, indicated that bonding social capital was inversely associated with climate anxiety. This finding could be reflective of the “buffering” or protective potential of bonding social capital and may align with the findings of a previous study by Hawkins and Maurer [29], which suggested that bonding social capital, compared with bridging and linking social capital, may more effectively foster immediate support after a hurricane. As such, in our study, bonding social capital was negatively associated with anxieties about climate change for our participants who were recovering from Hurricane Helene/Milton. On the other hand, we found that both bridging and linking social capital were associated with heightened climate anxiety. We proffer one possible explanation for these results. Specifically, it could be that participants’ heterogenous social ties, facilitated by bridging and linking social capital, may have comprised a large proportion of people expressing worries about the climate crisis. As such, this may have increased the likelihood of developing similar climate concerns. However, our surveys did not contain measures assessing the proportions of people expressing worries about the climate crisis within one’s heterogenous networks. We thus recommend future studies be conducted to measure this variable and examine its potential role in explaining how bridging and linking social capital could be both positively associated with climate anxiety.

The third purpose of our study was to explore the possibility that social capital would moderate the positive relationship between hurricane stressors and climate anxiety. To this end, our findings indicated that social capital did moderate the relationship between hurricane stressors and climate anxiety. However, the specific moderation pattern differed across bonding versus bridging and linking social capital. Specifically, our results suggested that bonding social capital’s protective role is conditional and reverses under high stress, rather than following a classic buffering pattern [43]. As Fig 1 shows, participants with high bonding social capital reported lower climate anxiety at low stressor levels. A classic buffering pattern would reveal a flattening hurricane stressor-anxiety slope for the high-bonding social capital group and steeper slopes for the low and moderate groups. Yet, our data showed the opposite. The slope was steeper for the high bonding social capital group. That is, bonding social capital weakens the relationship between hurricane stressors and climate anxiety only when hurricane stressors were low. As stressors accumulated, bonding social capital strengthens the association between stressors and climate anxiety, possibly because stress was transmitted within dense social networks [31]. Resources that are typically protective become less effective under high stress conditions [43]. In the context of hurricane coping and recovery, close-knit networks of family and friends often provide critical support in the early stages of a disaster [23]. However, when stressors accumulate, such as when hurricanes bring prolonged, repeated disruptions, the same networks may shift from being a source of support to a source of strain. For example, anxiety and worry may spread more easily within tightly knit groups, creating collective stress as shared resources become depleted. In addition, individuals embedded in strong bonding networks may feel the pressure to reciprocate by caring for others, which can amplify their own feelings of anxiety and emotional fatigue.

Yet our pattern of findings for bridging and linking social capital suggested that they amplified the positive association between hurricanes stressors and climate anxiety. This finding is in contrast with previous research that indicated social capital to serve as a buffer or play a protective role against anxiety following disasters [11]. It is possible that higher bridging and linking social capital increases the potential of being exposed to a wider span of information regarding climate change, namely, those pertaining to climate threats. As we noted, past research has found that exposure to information about the impacts of climate change is positively associated with climate anxiety [16]. One practical implication of these findings is that risk communication delivered through bridging and linking networks is better designed to pair disaster information with concrete efficacy and coping resources, so that heightened awareness does not only translate into distress. As the association between hurricane stressors and climate anxiety was strongest among individuals with high linking social capital, governmental and institutional risk communicators, in particular, may wish to attend to the social or emotional—not only the informational—consequences of their messages following disaster events.

Nevertheless, these findings collectively contribute to the literature by suggesting that the relationship between social capital and climate anxiety is more nuanced and there may be different theoretical mechanisms at play when assessing the role of social capital in one’s disaster experience. It further underscores the complexity of social capital’s role in different disaster scenarios. Furthermore, the just mentioned findings regarding social capital may also be reflective of the cognitive and structural dimensions of social capital. Its cognitive aspect focuses on trust and support perceptions, and they have been consistently linked to lowered anxiety and improved mental health state. However, studies have found that the structural dimension of social capital, indicated by one’s participation or connection to social networks, are related to mental health outcomes in more ambiguous ways. For example, Wind and colleagues [44] found that cognitive social capital was negatively related to mental health issues following a disaster, whereas structural social capital exacerbated feelings of anxiety. As the current operationalizations of social capital did not distinguish the cognitive and structural dimensions, the observed moderation relationship may be explained by the mixed effects of cognitive versus structural aspects of social capital.

Finally, although they were included as covariates in our models, we again note the following findings. First, the measure for generalized anxiety had a stronger association with climate anxiety than hurricane stressors did. The simple explanation for this is that generalized anxiety and climate anxiety are both anxiety constructs and likely share underlying features, even though they remain both conceptually distinct in their specific focus or content. On the other hand, hurricane stressors are an external, event-based exposure variable, and as such, are less proximate to an internalizing symptom measure like climate anxiety. Second, income and black race/ethnicity (b around.22) were both positively associated with climate anxiety. These findings respectively imply that individuals with higher household incomes and those who identify as black may experience greater climate anxiety. We speculate that higher-income individuals may be disproportionately exposed to climate-related information, which may heighten their climate anxiety. Additionally, the well-documented vulnerability of black communities to the adverse effects of climate-related disasters such as hurricanes [45] may similarly heighten their awareness of and anxiety about climate change. This warrants further investigation about potential racial and ethnic differences in climate anxiety. Finally, living in South Carolina was negatively associated with climate anxiety. One plausible explanation for this finding is the differential impact of Hurricanes Helene and Milton across states. While South Carolina was affected by Hurricane Helene, the storm’s most severe impacts were concentrated elsewhere. Thus, it could be that the South Carolina residents sampled in our study may have experienced comparatively less devastation, which may in turn have produced lower levels of climate anxiety. Future research could examine this explanation by including state-level measures that account for differential hurricane impacts.

### 5.1 Limitations and future directions

Although our study provides interesting results regarding experiencing an environmental disaster on people’s information behavior strategies, it has weaknesses that must be acknowledged. First, our study utilizes cross-sectional data. As a result, the cross-sectional design limits causal inference about whether social capital patterns preceded or resulted from hurricane exposure. Thus, we recommend future research, such as experimental studies be conducted to test whether in fact hurricane stressors are positively predictive of climate anxiety.

Second, potential discrepancies in our measures may have influenced our findings. Specifically, while our primary independent variable focuses on hurricane-related stressors, the corresponding measure of anxiety focuses on the issue of climate change more broadly. The issue here is that people may not directly associate hurricane experiences with climate change-specific anxiety. To address this issue, future studies should examine general anxiety tied to the specific event, to ensure a more precise match between the disaster experienced and the anxiety measured.

Third, regarding the measure used for climate change anxiety, we acknowledge that two of the three items drawn from Clayton and Karazsia’s climate anxiety scale [9] were taken from the scale’s functional impairment subscale, while one was drawn from the cognitive-emotional subscale. It could be argued, therefore, that these items more precisely captured climate change-related preoccupation or functional impairment, constructs that are related to but distinct from core climate symptomology. We note, however, that this limitation does not invalidate our findings, but rather contextualizes the interpretation of our outcome variable. Specifically, the climate change anxiety measure used in our study should be understood as reflecting climate change-related cognitive preoccupation and functional impairment rather than climate anxiety in its fullest sense. Ultimately, we recommend that future research use the full measure of climate change anxiety, which can adequately capture both dimensions of climate anxiety conceptualized by Clayton and Karazsia (i.e., functional impairment and cognitive-emotional dimensions) [9].

Fourth, while the Cronbach’s alpha for linking social capital in our study (α = .66) fell slightly below the conventional.70 threshold, this likely reflects the inherent multi-dimensionality of linking social capital as a construct rather than poor reliability per se. Cronbach’s alpha assumes unidimensionality [46]. This is a condition that works against linking social capital, which captures structurally distinct ties between individuals and institutional actors [22]. Yet we should note that the mean inter-item correlation for the linking social items in our present study was.41. This falls within the acceptable range recommended for multidimensional constructs [47], thus providing supplementary evidence of adequate reliability.

Finally, the data analyzed for this study were not representative of the population of interest. Nevertheless, we believe the use of a non-probability sample is appropriate given the context. Identifying a representative sample of individuals affected by hurricanes poses significant challenges, particularly when many potential participants are engaged in recovery efforts or may lack the time or resources to participate in the current research. Thus, even though these are not representative data, we believe it would be difficult to obtain this type of sample given the situation after the hurricanes.

### 5.2 Conclusion

Our findings help extend the stress process model and social capital research by showing the more nuanced role of social capital. Specifically, we extended understanding of the link between hurricane-related stressors among those who have experienced major hurricanes and their levels of climate change anxiety, and the role of social capital in conditioning this link. We also found some evidence suggesting that bonding social capital’s protective role is conditional and reverses under high stress. That is, bonding social capital may serve a protective role only when hurricane stressors are low, but not when they are high. Furthermore, rather than acting as a protective buffer, our study suggested higher levels of bridging and linking social capital were associated with more climate anxiety. While these findings for social capital may be viewed as more reflective of its “dark side” potential [16], it is worth noting that research suggests climate anxiety is also associated with adaptive coping strategies, such as increased engagement in pro-environmental behaviors and other actions to address or mitigate climate change [48,49]. As such, in cases where social capital strengthens the relationship between hurricane stressors and climate anxiety, higher levels of climate anxiety may result in adaptive climate-related actions, including greater engagement in pro-environmental and climate advocacy behaviors. Since our study did not assess climate anxiety’s impact on behaviors, it is important for research to further analyze this possibility. Practically, we should consider strengthening close social support and basic coping skills before disasters occur and stress escalates. Post-disaster efforts should manage information flows with clear updates, specific resources, and tips to help those in need, and counter misinformation to limit unnecessary anxiety.
