# Supplementary material for: Associations between hurricane stressors, social capital, and climate anxiety among individuals who experienced Hurricanes Helene and Milton
Source: PLOS Ment Health. 2026 Oct 13;3(10):e0000749. doi: 10.1371/journal.pmen.0000749 (PMC13660876; doi:10.1371/journal.pmen.0000749)
Supplement: Supplementary file 1 [file pmen.0000749.s002.docx]

**College of Journalism and Communications**	1885 Stadium Rd.

STEM Translational Communication Center	PO Box 118400

Gainesville, FL

32611

352-392-996

Journal Requirements:

 1. We have noticed that you have uploaded Supporting Information files, but you have not included a list of legends. Please add a full list of legends for your Supporting Information files after the references list.

**Response: We have now included legends on our References list.**

If the reviewer comments include a recommendation to cite specific previously published works, please review and evaluate these publications to determine whether they are relevant and should be cited. There is no requirement to cite these works unless the editor has indicated otherwise.

**Response: We have added references in order to respond to the Reviewers’ comments/suggestions. These additional references are highlighted.**

---------------

**Comments to the Author**

1. Does this manuscript meet PLOS Mental Health’s [publication criteria](https://nam10.safelinks.protection.outlook.com/?url=https%3A%2F%2Ftrack.editorialmanager.com%2FCL0%2Fhttps%3A%252F%252Fjournals.plos.org%252Fmentalhealth%252Fs%252Fcriteria-for-publication%2F1%2F010f019d1ec90e43-7be3596b-40f5-4975-9a54-d76066a59811-000000%2FFkxnODHBErqslcfOXo0_m8GdeZsUXEA_-SLbBRpADFs%3D255&data=05%7C02%7Cf.dalisay%40ufl.edu%7Cd4943e947eca435a7e8f08de89a76e0c%7C0d4da0f84a314d76ace60a62331e1b84%7C0%7C0%7C639099549404396321%7CUnknown%7CTWFpbGZsb3d8eyJFbXB0eU1hcGkiOnRydWUsIlYiOiIwLjAuMDAwMCIsIlAiOiJXaW4zMiIsIkFOIjoiTWFpbCIsIldUIjoyfQ%3D%3D%7C0%7C%7C%7C&sdata=AEhCN1plR7qxRl1EhzkMxlgXo9VFwLf6cLL7C3ttQLg%3D&reserved=0)? Is the manuscript technically sound, and do the data support the conclusions? The manuscript must describe methodologically and ethically rigorous research with conclusions that are appropriately drawn based on the data presented.

Reviewer #1: Yes

Reviewer #2: Partly

Reviewer #3: Yes

2. Has the statistical analysis been performed appropriately and rigorously?

Reviewer #1: Yes

Reviewer #2: Yes

Reviewer #3: Yes

3. Have the authors made all data underlying the findings in their manuscript fully available (please refer to the Data Availability Statement at the start of the manuscript PDF file)?

The [PLOS Data policy](https://nam10.safelinks.protection.outlook.com/?url=https%3A%2F%2Ftrack.editorialmanager.com%2FCL0%2Fhttps%3A%252F%252Fjournals.plos.org%252Fmentalhealth%252Fs%252Fdata-availability%2F1%2F010f019d1ec90e43-7be3596b-40f5-4975-9a54-d76066a59811-000000%2FU9RvibvUAgFqJxzBCcQ87pdnDR0AsOj3GDpCVz6nJHI%3D255&data=05%7C02%7Cf.dalisay%40ufl.edu%7Cd4943e947eca435a7e8f08de89a76e0c%7C0d4da0f84a314d76ace60a62331e1b84%7C0%7C0%7C639099549404406233%7CUnknown%7CTWFpbGZsb3d8eyJFbXB0eU1hcGkiOnRydWUsIlYiOiIwLjAuMDAwMCIsIlAiOiJXaW4zMiIsIkFOIjoiTWFpbCIsIldUIjoyfQ%3D%3D%7C0%7C%7C%7C&sdata=fOh4l93T36Cw6iPq6ZwmTDGTsgDJmhq6epuUkmKcRPc%3D&reserved=0) requires authors to make all data underlying the findings described in their manuscript fully available without restriction, with rare exception. The data should be provided as part of the manuscript or its supporting information, or deposited to a public repository. For example, in addition to summary statistics, the data points behind means, medians and variance measures should be available. If there are restrictions on publicly sharing data—e.g. participant privacy or use of data from a third party—those must be specified.

Reviewer #1: Yes

Reviewer #2: Yes

Reviewer #3: Yes

4. Is the manuscript presented in an intelligible fashion and written in standard English?

PLOS Mental Health does not copyedit accepted manuscripts, so the language in submitted articles must be clear, correct, and unambiguous. Any typographical or grammatical errors should be corrected at revision, so please note any specific errors here.

Reviewer #1: Yes

Reviewer #2: Yes

Reviewer #3: Yes

5. Review Comments to the Author

Please use the space provided to explain your answers to the questions above. You may also include additional comments for the author, including concerns about dual publication, research ethics, or publication ethics. (Please upload your review as an attachment if it exceeds 20,000 characters)

Reviewer #1: The manuscript is well aligned with the scope of the journal. It addresses mental health and well-being in the context of climate-related disasters and examines psychosocial resources (social capital) as both risk and protective factors. The focus on climate anxiety, disaster exposure, and social capital directly fits areas such as environmental impacts on mental health, socio-economic aspects, and well-being. Overall, the data support the main conclusions, and the authors are largely careful not to overstate their findings.

**Response: We thank you appreciate your review and comments and suggestions.**

That said, several revisions are recommended to improve clarity, coherence, and interpretability.
1. The cross-sectional design precludes any causal inference. While the authors generally avoid causal language, some interpretations (e.g., buffering versus amplifying effects) could be more cautiously framed as conditional associations rather than effects.

**Response: We have revised the manuscript to avoid causal language, such as “buffering” and “amplifying,” that implies causality in our interpretations of the conditional associations. Instead, we have phrase these more as associations – e.g., the relationship between hurricane stressors and climate change was stronger (or more pronounced, noticeable, etc.) with higher levels of social capital.**

2. Some reliability coefficients, particularly for linking social capital (α = .66), are marginal. While not unacceptable, this limitation should be acknowledged when interpreting findings involving this construct.

**Response: We have acknowledged this as a limitation. We have provided a more thorough response to a similar comment made by Reviewer 2.**

3. The variable climate change anxiety should be differenciated from the psychological construct anxiety, it is not well meassured for some of the conclusions.

**Response: In response to this comment and those of Reviewers 2 and 3, which touched on a similar concern, we have included additional content to conceptually distinguish climate change anxiety from generalized anxiety. As explained below, we have also included a measure of generalized anxiety as a covariate on our regression models.**

4. Table 1 introduces the variable Disaster Critical Thinking; however, this construct is not sufficiently described or theoretically framed in the main text. There is no clear definition, conceptual background, or justification for its inclusion provided in the introduction or methods sections. As a result, readers are left without an understanding of how Disaster Critical Thinking is conceptualized, how it differs from related constructs (e.g., risk perception, disaster preparedness, cognitive appraisal), or why it is relevant to climate anxiety within the proposed theoretical framework. To improve clarity and interpretability, the authors should: Explicitly define Disaster Critical Thinking in the text. Explain its theoretical grounding and relevance to disaster-related mental health outcomes. Describe how it was operationalized and measured before presenting it in Table 1.

**Response: We apologize for this oversight. We used a table from another manuscript of ours as a template to create Table 1. The variable, Disaster Critical Thinking, was mistakenly included in Table 1 and has since been deleted.**

5. Figures 1, 2, and 3 present interaction effects between hurricane stressors and different forms of social capital. While the figures are visually clear, a legend or description of the figures are needed to adequately explain the meaning of the colored lines displayed in each figure. Specifically, the text does not explicitly state which colors correspond to low, mean, or high levels (e.g., −1 SD, mean, +1 SD) of the moderator variables.

**Response: We have re-created the figures so that they provide more details that could help to adequately explain the meaning of the lines displayed in each figure.**

-----------------------------------------------------------------

Reviewer #2: Abstract

The abstract reads as though bonding capital is protective, when in fact its protective role is conditional and reverses under high stress.

**Response: Thanks for pointing this out. We have revised the abstract to specify that our results indicated that bonding social capital’s protective role is conditional and reverses under high stress. That is, bonding social capital weakens the relationship between hurricane stressors and climate anxiety only when hurricane stressors were low. As stressors accumulated, bonding social capital strengthens the association between stressors and climate anxiety.**

Introduction

The authors should more explicitly explain the cognitive pathway by which a hurricane experience activates concern about climate change rather than generalized distress.

**Response: Thanks much for this suggestion. On the Literature Review, as we discuss on subsequent responses below, we have provided content to more explicitly explain the cognitive pathway by which a hurricane experience activates concern about climate change rather than generalized distress.**

**Also, on the Introduction (page 4, line 53), on the following sentence, we have added that climate anxiety is “a form of distress distinct from generalized anxiety”: “Prior research particularly demonstrates that individuals who experience hurricane stressors—such as personal losses, injuries or other disruptions—may face an increased risk of developing climate anxiety [8], a form of distress distinct from generalized anxiety and defined as emotional or psychological distress marked by heightened fear and constant worrying about the climate crisis [9].”**

Methods

- The authors themselves acknowledge in the limitations section that hurricane stressors and climate change anxiety may not conceptually align well. This is not just a limitation, rather it poses a validity issue. The authors should either provide stronger theoretical justification for why hurricane exposure would activate climate-specific anxiety rather than general anxiety, or they should have added a general anxiety measure as a comparison outcome.

**Response: Thank you very much for this comment.**

**To address these issues, first, we have strengthened the theoretical justification for why hurricane exposure would activate climate-specific anxiety rather than generalized anxiety (see pages 5-6 of the revised manuscript). We did this by including Clayton’s (2003) framework of environmental identity and the concept of ecological grief (Cunsolo & Ellis, 2018), which was suggested by Reviewer 2.**

**Second, as our survey included a measure of generalized anxiety, we have now included a measure of generalized anxiety as a covariate in our regression models, which included four items adapted from the GAD-7 (Spitzer et al., 2006). The description of the measure and items used are now reported on page 7 of the revised manuscript. We have also revised the results reported on Tables 2 and 3 and Figures 1, 2, and 3 to reflect the addition of generalized anxiety as a covariate. By including this new covariate, our findings for the test of the hypothesis and investigation of the research questions remained consistent. The key exception is that at low levels of social capital (i.e., 1 SD below the mean), none of the three dimensions of social capital (bonding, bridging, and linking social capital) were significant moderators of the relationship between hurricane stressors and climate anxiety. However, all three dimensions of social capital remained significant moderators at the mean and at high levels of social capital (i.e., 1 SD above the mean).**

**Yet it is important that we note that the focus of our manuscript was not on empirically distinguishing generalized anxiety from climate change anxiety, as this would be beyond the scope of a single manuscript. Thus, we do not feel it is appropriate to examine generalized anxiety as an outcome for this present manuscript. Including it as a covariate should address Reviewer 1’s concern.**

**Clayton S. Environmental identity: A conceptual and an operational definition. Identity and the natural environment: The psychological significance of nature. 2003 Nov 7:45-65.**

**Cunsolo A, Ellis NR. Ecological grief as a mental health response to climate change-related loss. Nat Clim Change. 2018;8:275–81.**

**Spitzer RL, Kroenke K, Williams JBW, Löwe B. A brief measure for assessing generalized anxiety disorder: the GAD-7. Arch Intern Med. 2006; 166(10): 1092-7.**

- The alpha of linking social capital is below conventional .70 threshold. The authors need to justify this.

**Response: As noted in our response to a similar comment from Reviewer 1, we acknowledge this as a limitation (see pages 22-23 of the revised manuscript). While the Cronbach’s alpha for linking social capital in our study (α = .66) fell slightly below the conventional .70 threshold, this likely reflects the inherent multi-dimensionality of linking social capital as a construct rather than poor reliability per se. Cronbach’s alpha assumes unidimensionality [Cortina, 1993]. This is a condition that works against linking social capital, which captures structurally distinct ties between individuals and institutional actors [22, Portinga, 2012]. Yet we should note that the mean inter-item correlation for the linking social items on our present study was .41. This falls within the acceptable range recommended for multidimensional constructs [Briggs & Cheeks, 1986], thus providing supplementary evidence of adequate reliability.**

**Briggs SR, Cheek JM. The role of factor analysis in the development and evaluation of personality scales. J Pers. 1986;54(1):106–48.**

**Cortina JM. What is coefficient alpha? An examination of theory and applications. J Appl Psychol. 1993;78(1):98–104.**

Results

- There's a logical inconsistency in the final paragraph regarding which type of social capital is being discussed (Line 324 & 325). Please correctly explain.

**Response: We thank Reviewer 1 for pointing this out. We have now corrected the type of social capital from bridging to linking.**

- From table 3 we can see that even at 1 SD below the mean on bonding capital, the hurricane stressor–anxiety relationship remains positive and significant. This means bonding capital does not truly buffer, it only slows the strengthening of the relationship. The interpretation should be revised accordingly.

**Response: We have revised this interpretation both on the abstract (as requested by Reviewer 1) and on the Discussion section (see page 17). We should note (as was mentioned on a response to a previous comment), that after including generalized anxiety as a covariate, bonding social capital was no longer a significant moderator of the relationship between hurricane stressors and climate anxiety at low levels of bonding social capital (i.e., 1 SD below the mean).**

Other issues

- The paper refers to Figure 4 on line 319 but only Figures 1, 2 & 3 appears in the manuscript.

**Response: We thank Reviewer 3 for pointing this out. The correct figure referenced should have been “Figure 3,” which we have now corrected.**

- Table 1 includes Disaster critical thinking (M = 3.61, α = .86) which is never mentioned in the Methods or Results sections. This variable should either be explained or removed.

**Response: We have removed Disaster Critical Thinking from Table 1.**

-----------------------------------------------------------------

Reviewer #3: Major Strengths:
1. The study is timely and relevant.
2. There is a large sample size.
3. There is a strong conceptual contribution, showing that social capital is not always protective.

**Response: We thank Reviewer 3 for the above comments and we are very much appreciative for their review.**

Major Weaknesses:
There might be conceptual mismatch.

There might be underlying concepts behind hurricane stressors to suggest that it might be relate to climate change anxiety. Further, hurricane stressors does not automatically suggest that they are thinking about climate change. For climate change anxiety to arise, they must attribute it to climate change. Some people may attribute hurricanes to disasters rather than climate change. Perhaps, this could be added to limitations as well. Further studies may include climate risk perception or climate change attribution.

**Response: The central issues reflected on the comments directly above (i.e., underlying concepts, attribution of hurricanes to disasters rather than climate change) overlap with comments made by Reviewers 1 & 2. We believe these comments can be addressed by what Reviewer 1 had suggested in terms of strengthening the theoretical justification for why hurricane exposure would activate climate-specific anxiety rather than generalized anxiety. Specifically, we have incorporated Clayton’s environmental identity framework (2003) and the concept of ecological grief (Cunsolo & Ellis, 2018) to strengthen the theoretical justification, which was suggested by Reviewer 3 on the subsequent comment below.**

**Also, we have acknowledged as a limitation that our survey did not include measures for environmental identity, ecological grief, climate risk perceptions, or climate change attributions. We have noted that future studies could examine these variables as potential mechanisms that may explain the association between hurricane stressors and climate anxiety.**

2. The theoretical grounding is stress process model, when thinking about CCA, better explanations of theories includes Environmental Identity and other ecological grief models.

**Response: We thank Reviewer 3 very much for presenting environmental identity and the concept of ecological grief as frameworks to complement the stress process model. As explained above, we have now included these frameworks to further strengthen the theoretical explanation as to why hurricane stressors are associated with climate anxiety.**

3. Only three items were used from Clayton's CCA scale. The original scale has multiple dimensions, the chosen items may just indicate rumination not really anxiety, particularly CCA.

**Response: We appreciate this important methodological comment. We acknowledge that two of the three items used from Clayton’s Climate Anxiety Scale were drawn from the scale’s functional impairment subscale and one was drawn for its cognitive-emotional subscale. As Reviewer 3 noted, these items more precisely capture climate change-related cognitive preoccupation and functional impairment. These are related to but distinct from core climate anxiety symptomatology. On our revised manuscript, we have explicitly acknowledged this as a limitation and we now recommend that future studies employ the full scale, particularly the cognitive-emotional subscale (see page 22 of the revised manuscript). This would more comprehensively operationalize CCA. However, we believe this limitation does not invalidate our findings but rather qualifies the interpretation of our outcome variable, which should be understood as reflecting climate change-related cognitive preoccupation and functional impairment rather than climate anxiety in its fullest sense.**

4. Moderating results is interesting but can you explain the implications in more detail.

**Response: We feel we have provided sufficient implications regarding the moderating results on the first draft of the manuscript. However, we have added a few more details explaining the implications of the moderation results.**

Figure Resubmissions:

While revising your submission, we strongly recommend that you use PLOS’s NAAS tool ([https://ngplosjournals.pagemajik.ai/artanalysis](https://nam10.safelinks.protection.outlook.com/?url=https%3A%2F%2Fngplosjournals.pagemajik.ai%2Fartanalysis&data=05%7C02%7Cf.dalisay%40ufl.edu%7Cd4943e947eca435a7e8f08de89a76e0c%7C0d4da0f84a314d76ace60a62331e1b84%7C0%7C0%7C639099549404443219%7CUnknown%7CTWFpbGZsb3d8eyJFbXB0eU1hcGkiOnRydWUsIlYiOiIwLjAuMDAwMCIsIlAiOiJXaW4zMiIsIkFOIjoiTWFpbCIsIldUIjoyfQ%3D%3D%7C0%7C%7C%7C&sdata=fWbyRrYPxMHecxkPpFiK6%2B2eSA3Qk84TdKd4K7gwjsc%3D&reserved=0)) to test your figure files. NAAS can convert your figure files to the TIFF file type and meet basic requirements (such as print size, resolution), or provide you with a report on issues that do not meet our requirements and that NAAS cannot fix.
After uploading your figures to PLOS’s NAAS tool - [https://ngplosjournals.pagemajik.ai/artanalysis](https://nam10.safelinks.protection.outlook.com/?url=https%3A%2F%2Fngplosjournals.pagemajik.ai%2Fartanalysis&data=05%7C02%7Cf.dalisay%40ufl.edu%7Cd4943e947eca435a7e8f08de89a76e0c%7C0d4da0f84a314d76ace60a62331e1b84%7C0%7C0%7C639099549404454656%7CUnknown%7CTWFpbGZsb3d8eyJFbXB0eU1hcGkiOnRydWUsIlYiOiIwLjAuMDAwMCIsIlAiOiJXaW4zMiIsIkFOIjoiTWFpbCIsIldUIjoyfQ%3D%3D%7C0%7C%7C%7C&sdata=RnhKbXiMMgu%2Fa1zb8RLYg9IJk6MQKMf%2BYuYBgsXDQJc%3D&reserved=0), NAAS will process the files provided and display the results in the "Uploaded Files" section of the page as the processing is complete. If the uploaded figures meet our requirements (or NAAS is able to fix the files to meet our requirements), the figure will be marked as "fixed" above. If NAAS is unable to fix the files, a red "failed" label will appear above. When NAAS has confirmed that the figure files meet our requirements, please download the file via the download option, and include these NAAS processed figure files when submitting your revised manuscript.

**Response: We have used PLOS’s NAAS tool to prepare the figures.**
