# Supplementary material for: Associations between hurricane stressors, social capital, and climate anxiety among individuals who experienced Hurricanes Helene and Milton
Source: PLOS Ment Health. 2026 Oct 13;3(10):e0000749. doi: 10.1371/journal.pmen.0000749 (PMC13660876; doi:10.1371/journal.pmen.0000749)
Supplement: Supplementary file 2 [file pmen.0000749.s003.docx]

**Response to Reviewers**

**Reviewer #1:** Thank you for your careful revision of the manuscript. The revised version successfully addresses the reviewers' comments and demonstrates substantial improvements in clarity, coherence, and overall presentation. The study is well aligned with the scope of the journal, addressing an important and timely topic in mental health with a more clear theoretical framework, appropriate methodology, and conclusions that are well supported by the data. These revisions have significantly strengthened the manuscript, and I believe it is now suitable for publication and will make a valuable contribution to the literature on climate-related mental health and disaster recovery.

**Reviewer #3:** Congratulations!

**Response: We thank the reviewers very much for their time and commitment in reviewing our manuscript. Since Reviewer #2 provided additional comments, we address them below.**

--

**Reviewer #2:** This is a substantive effort to address prior concerns.

Abstract
Add one clause noting that associations held after controlling for GAD-7 as it was one of the largest and most robust predictors of the model to help readers easily understand the findings.

**Response: We have added the following clause to the abstract that notes that the focal associations held after controlling for symptoms of generalized anxiety (see page 2): “…and these associations held after controlling for symptoms of generalized anxiety.”**

Introduction & Literature Review
These sections read well, no major concern from my side.

**Response: We thank you very much for your comment.**

Materials and Methods
Only 4 of the 7 standard GAD-7 items were used, and no rationale or reliability statistic is reported. Given how influential GAD-7 in Table 2, this omission is now more consequential.

Report reliability for the 4-item measure and briefly justify the subset.

**Response: We should first clarify that the use of four items from the GAD-7 is well-supported by precedent in the literature. Notably, GAD-2, a two-item subset of GAD-7, has been validated as a reliable screening measure across multiple settings (see Kroenke et al., 2007). As a two-item subset is considered psychometrically defensible, a four-item subset is at minimum equally justified, as it captures a broader range of generalized anxiety symptomology while remaining parsimonious. Moreover, we note that we did not report reliability for the generalized anxiety measure because it was included as a covariate rather than a focal variable in the study. However, to address the reviewer’s concern, under the section “3.3.4 Covariates” (see page 14), we have now reported the Cronbach’s alpha for the four items adapted from the GAD-7 (α = .93). This reflects the reliability and addresses the reviewer's concern about the absence of a reliability statistic.**

**Reference:**

**Kroenke K, Spitzer RL, Williams JB, Monahan PO, Löwe B. Anxiety disorders in primary care: prevalence, impairment, comorbidity, and detection. Ann Intern Med. 2007;146(5):317–25.**

With GAD-7 and climate anxiety both measured, reporting their bivariate correlation becomes essential. If that correlation is high, it raises a multicollinearity/construct-overlap question.

**Response: The bivariate correlation between the four-item generalized anxiety index and climate anxiety is r = .24, p < .05, which we now report on page 15. This level of correlation does not raise concerns about multicollinearity.**

Results
Income (b around .054–.056) and Black race/ethnicity (b around .22) are both significant positive predictors of climate anxiety, and South Carolina residence is a significant negative predictor (b around -.23 to -.25) relative to the Florida reference group. None of these is mentioned anywhere in Results or Discussion.

**Response: We initially chose not to report these results, as they pertain to covariates and were not the focus of our study. However, to address the reviewer's comment, we have created a new sub-section with the sub-heading “4.3 Ancillary analyses: Association of covariates with climate anxiety,” and provided the following statement reporting the above findings: “As Table 2 shows, income (b around .054–.056) and black race/ethnicity (b around .22) were both positively associated with climate anxiety, and South Carolina residence (relative to the Florida reference group) was negatively associated (b around -.23 to -.25).”**

**In addition, in the Discussion section, we have now noted that the findings are noteworthy patterns warranting future investigation: “Finally, although they were included as covariate in our models, we again note the following findings. First, income and black race/ethnicity (b around .22) were both positively associated with climate anxiety. These findings respectively imply that individuals with higher household incomes and those who identify as Black may experience greater climate anxiety. We speculate that higher-income individuals may be disproportionately exposed to climate-related information, which may heighten their climate anxiety. Additionally, the well-documented vulnerability of Black communities to the adverse effects of climate-related disasters such as hurricanes [45] may similarly heighten their awareness of and anxiety about climate change. This warrants further investigation about potential racial and ethnic differences in climate anxiety. Finally, living in South Carolina was negatively associated with climate anxiety. One plausible explanation for this finding is the differential impact of Hurricanes Helene and Milton across states. While South Carolina was affected by Hurricane Helene, the storm’s most severe impacts were concentrated elsewhere. Thus, it could be that the South Carolina residents sampled in our study may have experienced comparatively less devastation, which may in turn have produced lower levels of climate anxiety. Future research could examine this explanation by including state-level measures that account for differential hurricane impacts.”**

**Reference:**

**45. Berberian AG, Gonzalez DJX, Cushing LJ. Racial disparities in climate change-related health effects in the United States. Curr Environ Health Rep. 2022;9(3):451-64. doi:10.1007/s40572-022-00360-w.**

The GAD-7's own effect size is large across all three models is more than double the effect size of hurricane stressor. This deserves explicit reporting and interpretation in Results.

**Response: We chose not to report the results for generalized anxiety (GAD-7), as it was merely a covariate and was not the focus of our study. However, to address the reviewer's comment, as noted above, we have created a new sub-section with the sub-heading “4.3 Ancillary analyses: Association of covariates with climate anxiety.” This section now provides a statement reporting the regression coefficients representing the association between generalized anxiety and climate anxiety, which are already reported in Table 2 of the manuscript: “Also, generalized anxiety as a covariate was positively associated with climate change anxiety (b = between -.178 to -.182).”**

Table 2 reports R² for the three models but there is no ΔR² comparing models with vs. without the GAD-7. A reader cannot tell how much unique variance generalized anxiety accounts for versus the focal predictors. Report ΔR² (or a hierarchical entry step) for the GAD-7 covariate specifically.

**Response: We appreciate this comment. We should note that the primary aim of the study was to examine associations between hurricane stressors, forms of social capital (bonding, bridging, linking), and climate anxiety, with generalized anxiety merely included as a covariate to account for its potential influence. That said, we agree that reporting its unique contribution adds transparency. Note too that the analyses were conducted using the PROCESS macro, which relies on OLS regression (Hayes, 2022). In this context, the unique variance explained by any predictor is captured by its squared semipartial correlation (sr²), the statistical significance of which corresponds directly to the t-statistic of its regression coefficient. Thus, rather than a hierarchical ΔR² approach, we report sr² for the GAD-7 covariate to quantify the unique variance it accounts for in climate anxiety after controlling for all other predictors in the model. In Table 2, we have now included sr² for each predictor, expressed as a percentage, reflecting the unique variance accounted for by each predictor.**

**Reference:**

**Hayes AF. Introduction to Mediation, Moderation, and Conditional Process Analysis: A Regression-Based Approach. 3rd ed. New York: Guilford Press; 2022.**

Discussion
GAD-7, Income, Racial disparity, State-level effect all of them need to be explicitly address in the Discussion section.

**Response: As noted above, we have provided a brief statement interpreting these findings on the Discussion section.**
