# Supplementary material for: Associations between hurricane stressors, social capital, and climate anxiety among individuals who experienced Hurricanes Helene and Milton
Source: PLOS Ment Health. 2026 Oct 13;3(10):e0000749. doi: 10.1371/journal.pmen.0000749 (PMC13660876; doi:10.1371/journal.pmen.0000749)
Supplement: Peer review history

# Peer review: Decision Letter 0

**DOI:** 10.1371/journal.pmen.0000749.r001

**Authors:** Marc Eric Reyes

24 Mar 2026

PMEN-D-25-00562

Associations Between Hurricane Stressors, Social Capital, and Climate Anxiety Among Individuals Who Experienced Hurricanes Helene and Milton

PLOS Mental Health

Dear Dr. Dalisay,

Thank you for submitting your manuscript to PLOS Mental Health. After careful consideration, we feel that it has merit but does not fully meet PLOS Mental Health’s publication criteria as it currently stands. Therefore, we invite you to submit a revised version of the manuscript that addresses the points raised during the review process.

Please submit your revised manuscript by May 08 2026 11:59PM. If you will need more time than this to complete your revisions, please reply to this message or contact the journal office at mentalhealth@plos.org. When you're ready to submit your revision, log on to https://www.editorialmanager.com/pmen/ and select the 'Submissions Needing Revision' folder to locate your manuscript file.

Please include the following items when submitting your revised manuscript:

- A letter that responds to each point raised by the editor and reviewer(s). You should upload this letter as a separate file labeled 'Response to Reviewers'.
- A marked-up copy of your manuscript that highlights changes made to the original version. You should upload this as a separate file labeled 'Revised Manuscript with Track Changes'.
- An unmarked version of your revised paper without tracked changes. You should upload this as a separate file labeled 'Manuscript'.

Guidelines for resubmitting your figure files are available below the reviewer comments at the end of this letter.

We look forward to receiving your revised manuscript.

Kind regards,

Marc Eric Santos Reyes

Academic Editor

PLOS Mental Health

Journal Requirements:

1. We have noticed that you have uploaded Supporting Information files, but you have not included a list of legends. Please add a full list of legends for your Supporting Information files after the references list.

If the reviewer comments include a recommendation to cite specific previously published works, please review and evaluate these publications to determine whether they are relevant and should be cited. There is no requirement to cite these works unless the editor has indicated otherwise.

Additional Editor Comments (if provided):

[Note: HTML markup is below. Please do not edit.]

Reviewers' comments:

Reviewer's Responses to Questions

**Comments to the Author**

1. Does this manuscript meet PLOS Mental Health’s publication criteria?

Reviewer #1: Yes

Reviewer #2: Partly

Reviewer #3: Yes

**********

2. Has the statistical analysis been performed appropriately and rigorously?

Reviewer #1: Yes

Reviewer #2: Yes

Reviewer #3: Yes

**********

3. Have the authors made all data underlying the findings in their manuscript fully available (please refer to the Data Availability Statement at the start of the manuscript PDF file)?

The PLOS Data policy

Reviewer #1: Yes

Reviewer #2: Yes

Reviewer #3: Yes

**********

4. Is the manuscript presented in an intelligible fashion and written in standard English?

Reviewer #1: Yes

Reviewer #2: Yes

Reviewer #3: Yes

**********

Reviewer #1: The manuscript is well aligned with the scope of the journal. It addresses mental health and well-being in the context of climate-related disasters and examines psychosocial resources (social capital) as both risk and protective factors. The focus on climate anxiety, disaster exposure, and social capital directly fits areas such as environmental impacts on mental health, socio-economic aspects, and well-being. Overall, the data support the main conclusions, and the authors are largely careful not to overstate their findings.

That said, several revisions are recommended to improve clarity, coherence, and interpretability.

1. The cross-sectional design precludes any causal inference. While the authors generally avoid causal language, some interpretations (e.g., buffering versus amplifying effects) could be more cautiously framed as conditional associations rather than effects.

2. Some reliability coefficients, particularly for linking social capital (α = .66), are marginal. While not unacceptable, this limitation should be acknowledged when interpreting findings involving this construct.

3. The variable climate change anxiety should be differenciated from the psychological construct anxiety, it is not well meassured for some of the conclusions.

4. Table 1 introduces the variable Disaster Critical Thinking; however, this construct is not sufficiently described or theoretically framed in the main text. There is no clear definition, conceptual background, or justification for its inclusion provided in the introduction or methods sections. As a result, readers are left without an understanding of how Disaster Critical Thinking is conceptualized, how it differs from related constructs (e.g., risk perception, disaster preparedness, cognitive appraisal), or why it is relevant to climate anxiety within the proposed theoretical framework. To improve clarity and interpretability, the authors should: Explicitly define Disaster Critical Thinking in the text. Explain its theoretical grounding and relevance to disaster-related mental health outcomes. Describe how it was operationalized and measured before presenting it in Table 1.

5. Figures 1, 2, and 3 present interaction effects between hurricane stressors and different forms of social capital. While the figures are visually clear, a legend or description of the figures are needed to adequately explain the meaning of the colored lines displayed in each figure. Specifically, the text does not explicitly state which colors correspond to low, mean, or high levels (e.g., −1 SD, mean, +1 SD) of the moderator variables.

Reviewer #2: Abstract

The abstract reads as though bonding capital is protective, when in fact its protective role is conditional and reverses under high stress.

Introduction

The authors should more explicitly explain the cognitive pathway by which a hurricane experience activates concern about climate change rather than generalized distress.

Methods

- The authors themselves acknowledge in the limitations section that hurricane stressors and climate change anxiety may not conceptually align well. This is not just a limitation, rather it poses a validity issue. The authors should either provide stronger theoretical justification for why hurricane exposure would activate climate-specific anxiety rather than general anxiety, or they should have added a general anxiety measure as a comparison outcome.

- The alpha of linking social capital is below conventional .70 threshold. The authors need to justify this.

Results

- There's a logical inconsistency in the final paragraph regarding which type of social capital is being discussed (Line 324 & 325). Please correctly explain.

- From table 3 we can see that even at 1 SD below the mean on bonding capital, the hurricane stressor–anxiety relationship remains positive and significant. This means bonding capital does not truly buffer, it only slows the strengthening of the relationship. The interpretation should be revised accordingly.

Other issues

- The paper refers to Figure 4 on line 319 but only Figures 1, 2 & 3 appears in the manuscript.

- Table 1 includes Disaster critical thinking (M = 3.61, α = .86) which is never mentioned in the Methods or Results sections. This variable should either be explained or removed.

Reviewer #3: Major Strengths:

1. The study is timely and relevant.

2. There is a large sample size.

3. There is a strong conceptual contribution, showing that social capital is not always protective.

Major Weaknesses:

1. There might be conceptual mismatch. There might be underlying concepts behind hurricane stressors to suggest that it might be relate to climate change anxiety. Further, hurricane stressors does not automatically suggest that they are thinking about climate change. For climate change anxiety to arise, they must attribute it to climate change. Some people may attribute hurricanes to disasters rather than climate change. Perhaps, this could be added to limitations as well. Further studies may include climate risk perception or climate change attribution.

2. The theoretical grounding is stress process model, when thinking about CCA, better explanations of theories includes Environmental Identity and other ecological grief models.

3. Only three items were used from Clayton's CCA scale. The original scale has multiple dimensions, the chosen items may just indicate rumination not really anxiety, particularly CCA.

4. Moderating results is interesting but can you explain the implications in more detail.

**********

what does this mean?). If published, this will include your full peer review and any attached files.

**Do you want your identity to be public for this peer review?**  If you choose “no”, your identity will remain anonymous but your review may still be made public.

For information about this choice, including consent withdrawal, please see our Privacy Policy

Reviewer #1: **Yes:**  Ruth Natalia Suarez Florez

Reviewer #2: No

Reviewer #3: No

**********

[NOTE: If reviewer comments were submitted as an attachment file, they will be attached to this email and accessible via the submission site. Please log into your account, locate the manuscript record, and check for the action link "View Attachments". If this link does not appear, there are no attachment files.]

# Author comment: Author response to Decision Letter 1

**DOI:** 10.1371/journal.pmen.0000749.r002

19 Jun 2026

Attachment Submitted filename: R&R.Climate Anxiety.Responses to Reviewers.docx

# Peer review: Decision Letter 1

**DOI:** 10.1371/journal.pmen.0000749.r003

**Authors:** Marc Eric Reyes

24 Aug 2026

PMEN-D-25-00562R1

Associations Between Hurricane Stressors, Social Capital, and Climate Anxiety Among Individuals Who Experienced Hurricanes Helene and Milton

PLOS Mental Health

Dear Dr. Dalisay,

Thank you for submitting your manuscript to PLOS Mental Health. After careful consideration, we feel that it has merit but does not fully meet PLOS Mental Health’s publication criteria as it currently stands. Therefore, we invite you to submit a revised version of the manuscript that addresses the points raised during the review process.

Please submit your revised manuscript by Sep 23 2026 11:59PM. If you will need more time than this to complete your revisions, please reply to this message or contact the journal office at mentalhealth@plos.org. When you're ready to submit your revision, log on to https://www.editorialmanager.com/pmen/ and select the 'Submissions Needing Revision' folder to locate your manuscript file.

Please include the following items when submitting your revised manuscript:

- A letter that responds to each point raised by the editor and reviewer(s). You should upload this letter as a separate file labeled 'Response to Reviewers'.
- A marked-up copy of your manuscript that highlights changes made to the original version. You should upload this as a separate file labeled 'Revised Manuscript with Track Changes'.
- An unmarked version of your revised paper without tracked changes. You should upload this as a separate file labeled 'Manuscript'.

Guidelines for resubmitting your figure files are available below the reviewer comments at the end of this letter.

As the corresponding author, your ORCID iD is verified in the submission system and will appear in the published article. PLOS supports the use of ORCID, and we encourage all coauthors to register for an ORCID iD and use it as well. Please encourage your coauthors to verify their ORCID iD within the submission system before final acceptance, as unverified ORCID iDs will not appear in the published article. *Only*  the individual author can complete the verification step; PLOS staff *cannot*  verify ORCID iDs on behalf of authors.

We look forward to receiving your revised manuscript.

Kind regards,

Marc Eric Santos Reyes

Academic Editor

PLOS Mental Health

**Journal Requirements** :

If the reviewer comments include a recommendation to cite specific previously published works, please review and evaluate these publications to determine whether they are relevant and should be cited. There is no requirement to cite these works unless the editor has indicated otherwise.

Please review your reference list to ensure that it is complete and correct. If you have cited papers that have been retracted, please include the rationale for doing so in the manuscript text, or remove these references and replace them with relevant current references. Any changes to the reference list should be mentioned in the rebuttal letter that accompanies your revised manuscript. If you need to cite a retracted article, indicate the article’s retracted status in the References list and also include a citation and full reference for the retraction notice.

**Additional Editor Comments (if provided):**

[Note: HTML markup is below. Please do not edit.]

Reviewers' comments:

Reviewer's Responses to Questions

**Comments to the Author**

Reviewer #1: All comments have been addressed

Reviewer #2: All comments have been addressed

Reviewer #3: All comments have been addressed

**********

publication criteria?

Reviewer #1: Yes

Reviewer #2: Yes

Reviewer #3: Yes

**********

3. Has the statistical analysis been performed appropriately and rigorously?

Reviewer #1: Yes

Reviewer #2: Yes

Reviewer #3: Yes

**********

4. Have the authors made all data underlying the findings in their manuscript fully available (please refer to the Data Availability Statement at the start of the manuscript PDF file)?

The PLOS Data policy

Reviewer #1: Yes

Reviewer #2: Yes

Reviewer #3: Yes

**********

5. Is the manuscript presented in an intelligible fashion and written in standard English?

Reviewer #1: Yes

Reviewer #2: Yes

Reviewer #3: Yes

**********

**Reviewer #1:**  Thank you for your careful revision of the manuscript. The revised version successfully addresses the reviewers' comments and demonstrates substantial improvements in clarity, coherence, and overall presentation. The study is well aligned with the scope of the journal, addressing an important and timely topic in mental health with a more clear theoretical framework, appropriate methodology, and conclusions that are well supported by the data. These revisions have significantly strengthened the manuscript, and I believe it is now suitable for publication and will make a valuable contribution to the literature on climate-related mental health and disaster recovery.

**Reviewer #2:**  This is a substantive effort to address prior concerns.

Here are my comments:

Abstract

Add one clause noting that associations held after controlling for GAD-7 as it was one of the largest and most robust predictors of the model to help readers easily understand the findings.

Introduction & Literature Review

These sections read well, no major concern from my side.

Materials and Methods

Only 4 of the 7 standard GAD-7 items were used, and no rationale or reliability statistic is reported. Given how influential GAD-7 in Table 2, this omission is now more consequential. Report reliability for the 4-item measure and briefly justify the subset.

With GAD-7 and climate anxiety both measured, reporting their bivariate correlation becomes essential. If that correlation is high, it raises a multicollinearity/construct-overlap question.

Results

Income (b around .054–.056) and Black race/ethnicity (b around .22) are both significant positive predictors of climate anxiety, and South Carolina residence is a significant negative predictor (b around -.23 to -.25) relative to the Florida reference group. None of these is mentioned anywhere in Results or Discussion.

The GAD-7's own effect size is large across all three models is more than double the effect size of hurricane stressor. This deserves explicit reporting and interpretation in Results.

Table 2 reports R² for the three models but there is no ΔR² comparing models with vs. without the GAD-7. A reader cannot tell how much unique variance generalized anxiety accounts for versus the focal predictors. Report ΔR² (or a hierarchical entry step) for the GAD-7 covariate specifically.

Discussion

GAD-7, Income, Racial disparity, State-level effect all of them need to be explicitly address in the Discussion section.

**Reviewer #3:**  Congratulations!

**********

what does this mean?). If published, this will include your full peer review and any attached files.

**Do you want your identity to be public for this peer review?**  If you choose “no”, your identity will remain anonymous but your review may still be made public.

For information about this choice, including consent withdrawal, please see our Privacy Policy

Reviewer #1: **Yes:**  Ruth Natalia Suárez Flórez

Reviewer #2: **Yes:**  Mahady Hasan Asif

Reviewer #3: **Yes:**  Alvin Joseph Mapoy

**********

[NOTE: If reviewer comments were submitted as an attachment file, they will be attached to this email and accessible via the submission site. Please log into your account, locate the manuscript record, and check for the action link "View Attachments". If this link does not appear, there are no attachment files.]

**Figure Resubmissions** :

# Author comment: Author response to Decision Letter 2

**DOI:** 10.1371/journal.pmen.0000749.r004

11 Sep 2026

Attachment Submitted filename: R&R.Responses to Reviewers.docx

# Editor report: Decision Letter 2

**DOI:** 10.1371/journal.pmen.0000749.r005

**Authors:** Marc Eric Reyes

24 Sep 2026

Associations Between Hurricane Stressors, Social Capital, and Climate Anxiety Among Individuals Who Experienced Hurricanes Helene and Milton

PMEN-D-25-00562R2

Dear Dr. Dalisay,

We are pleased to inform you that your manuscript 'Associations Between Hurricane Stressors, Social Capital, and Climate Anxiety Among Individuals Who Experienced Hurricanes Helene and Milton' has been provisionally accepted for publication in PLOS Mental Health.

Before your manuscript can be formally accepted you will need to complete some formatting changes, which you will receive in a follow up email. A member of our team will be in touch with a set of requests.

Please note that your manuscript will not be scheduled for publication until you have made the required changes, so a swift response is appreciated.

IMPORTANT: The editorial review process is now complete. PLOS will only permit corrections to spelling, formatting or significant scientific errors from this point onwards. Requests for major changes, or any which affect the scientific understanding of your work, will cause delays to the publication date of your manuscript.

If your institution or institutions have a press office, please notify them about your upcoming paper to help maximize its impact. If they'll be preparing press materials, please inform our press team as soon as possible -- no later than 48 hours after receiving the formal acceptance. Your manuscript will remain under strict press embargo until 2 pm Eastern Time on the date of publication. For more information, please contact mentalhealth@plos.org.

Thank you again for supporting Open Access publishing; we are looking forward to publishing your work in PLOS Mental Health.

Best regards,

Marc Eric Santos Reyes

Academic Editor

PLOS Mental Health

***********************************************************

Reviewer Comments (if any, and for reference):
